# Romosozumab in postmenopausal women with classical Osteogenesis imperfecta

**DOI:** 10.1007/s11657-026-01744-3

**Published:** 2026-07-18

**Authors:** Mikolaj Bartosik, Oskar Windels, Felix N. von Brackel, Michael Amling, Ralf Oheim

**Affiliations:** Department of Osteology and Biomechanics, University Medical Center Hamburg-Eppendorf, Lottestrasse 59, 22529 Hamburg, Germany

**Keywords:** Osteogenesis imperfecta, Romosozumab, HR-pQCT, DXA, Anti-sclerostin antibody

## Abstract

***Summary *:**

This study explored whether Romosozumab, an anti-sclerostin antibody, can improve bone quality in women with Osteogenesis imperfecta. After 12 months, spinal areal bone mineral density increased, showing a beneficial treatment effect, although changes in hip bone density and bone structure were less pronounced than in women with severe osteoporosis.

**Purpose:**

Osteogenesis imperfecta (OI) is the most common hereditary bone disorder, characterized by increased bone fragility and impaired bone quality, but pharmacological treatment is limited. Ongoing clinical trials investigate monoclonal anti-sclerostin antibodies for OI patients, offering new hope for reducing bone fragility by increasing bone mass.

**Methods:**

Postmenopausal women with either OI (*n* = 5) or severe osteoporosis (OPO, *n* = 10) receiving Romosozumab monthly (210 mg s.c.) for 12 months were analyzed retrospectively. Clinical assessments were performed at baseline, after 6 months, and after 12 months. Bone mass and structure were evaluated at baseline and after 12 months of treatment. In addition, serum and urine markers of bone turnover were analyzed at each time point.

**Results:**

The mean age of the participants was 53.6 ± 8.9 years for OI patients and 57.2 ± 5.8 years for OPO patients (*p* = 0.444). After 12 months of Romosozumab treatment, spinal aBMD and osteocalcin increased significantly in OI patients, indicating an anabolic response. HR-pQCT analysis revealed no statistically significant microstructural changes in patients with OI, although trends and moderate effect sizes suggested potential improvements. In patients with OPO, we observed a more pronounced response of bone turnover markers with greater aBMD gains at both the spine and femur, as well as significant improvements in bone microstructure, particularly at the tibia.

**Conclusions:**

Romosozumab treatment in postmenopausal women with OI resulted in a significant increase in spinal aBMD, though the effect on hip aBMD and peripheral bone microstructure was limited in contrast to postmenopausal osteoporosis.

## Introduction

Classical Osteogenesis imperfecta (OI) is the most common hereditary bone disease caused by pathogenic variants in the *COL1A1* or *COL1A2* genes, which encode the α-chains of type I collagen, an important structural component of the bone matrix. Therefore, patients carrying variants in these genes show reduced bone quality and increased bone fragility [1, 2]. According to Sillence et al. classical OI can be classified into four different subgroups I-IV [3]. Despite advances in disease understanding as well as supportive and medical care, the specific pharmacological treatment of adult OI patients improving bone mass and quality continues to be difficult, and treatment options are still very limited. Bisphosphonates remain the most commonly used treatment [4, 5], but anabolic agents such as teriparatide have also been studied in adult OI patients [5–7].

Sclerostin is a protein predominantly produced by osteocytes inhibiting the Wnt signaling pathway by binding to the low-density lipoprotein receptor-related proteins LRP5 and LRP6 on bone-forming cells [8, 9]. This leads to suppression of osteoblast differentiation and activity, thereby inhibiting bone formation. Additionally, sclerostin stimulates the expression of RANKL in osteocytes, leading to increased activation of osteoclasts and thus bone resorption [10]. The identification of the inhibitory role of sclerostin in bone metabolism has led to the development of therapeutic antibodies that neutralize its activity to increase bone formation [11, 12]. Romosozumab, a humanized monoclonal anti-sclerostin antibody, simultaneously stimulates bone formation and reduces bone resorption (to a lesser extend). It demonstrated significant increases in areal bone mineral density (aBMD) and a reduction in fracture risk in postmenopausal women with osteoporosis [13, 14]. Based on the aforementioned mechanisms, sclerostin inhibition may also be a promising therapeutic approach for the treatment of OI. Ongoing clinical trials (NCT05972551, NCT04545554, NCT05312697, NCT05768854, NCT05125809) are currently evaluating the safety and efficacy of anti-sclerostin antibodies in children, adolescents, and adult OI patients, with preliminary results suggesting improvements in bone mass and microstructure [15, 16].

Here, we investigated the treatment effect of twelve months of Romosozumab treatment on bone turnover markers, aBMD, and bone microstructure in postmenopausal OI patients with severe osteoporosis and vertebral fractures. Subsequently, we compared the treatment effects of postmenopausal OI patients with Romosozumab treated postmenopausal osteoporosis patients without OI.

## Methods

### Study design

This retrospective study was conducted in accordance with the local ethics committee (PV5364 and 2023–101124-BO-ff) and the Declaration of Helsinki. Inclusion criteria comprised postmenopausal status, severe osteoporosis for the OPO and OI group, as well as treatment with Romosozumab. Severe osteoporosis was defined as a lowest T-score ≤ −2.5 by DXA with at least one vertebral fragility fracture [17]. Exclusion criteria were other hereditary or metabolic bone diseases, or active bone tumors. A total of 5 adult postmenopausal women with OI were treated with Romosozumab for 12 months (210 mg subcutaneously, once monthly) in our specialized outpatient clinic. OI patients were clinically and genetically diagnosed with classical OI (either type I (*n* = 4) or IV (*n *= 1)). Additionally, ten age-matched postmenopausal women with severe osteoporosis receiving 12 months of Romosozumab (210 mg subcutaneously, once monthly) treatment, were included as a control group. Check-ups were performed at baseline (t0), after 6 months (t1), and after 12 months (t2). Patients underwent biochemical analyses, bone density measurements using dual-energy X-ray absorptiometry, and bone microstructure assessment using high resolution peripheral quantitative computed tomography as part of the clinical routine. Adverse drug events, number of vertebral and peripheral fractures were determined using a medical history questionnaire.

### Biochemical analysis

**Fig. 1:**
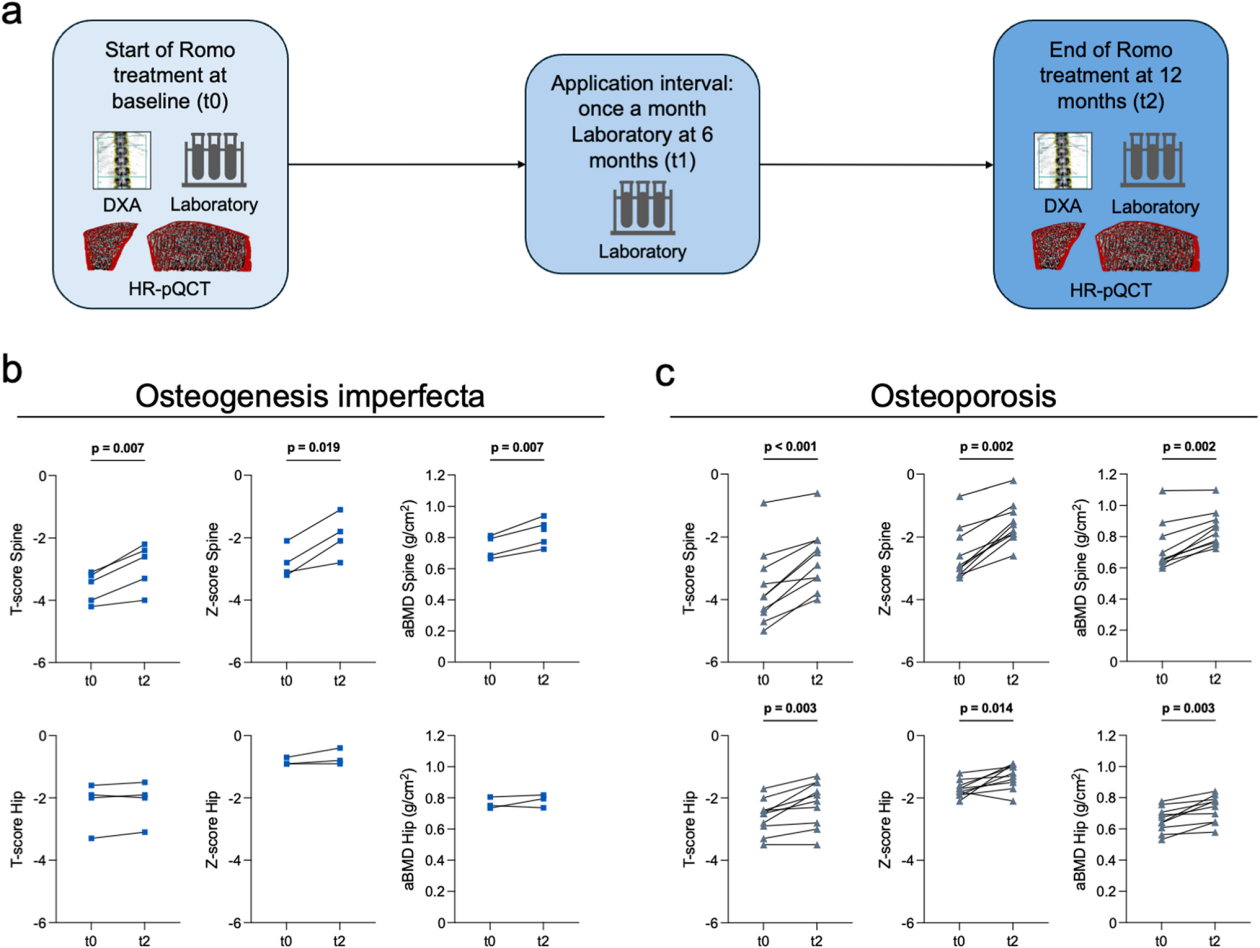
Bone mineral density measured by DXA in patients with postmenopausal OI and postmenopausal osteoporosis receiving Romosozumab treatment (OPO). DXA and HR-pQCT scans were performed at baseline or within the first year before Romosozumab treatment (t0) and after 12 months of treatment (t2) (**a**). Romosozumab was administered at monthly intervals, with laboratory assessments conducted at baseline (t0), after 6 months (t1), and after 12 months (t2) (**a**). The lowest T-score of two adjacent lumbar vertebrae, together with the corresponding Z-score and aBMD, as well as the lowest T-score of the total hip or femoral neck with corresponding parameters, were used for analysis. The OI group is shown in blue (**b**), and the OPO group in gray (**c**). One OI patient contributed data from an external measurement at t0, whereas hip assessment was unavailable in another OI patient due to the presence of metal implants. HR-pQCT: high-resolution peripheral quantitative computed tomography; Romo: Romosozumab; DXA: dual-energy X-ray absorptiometry; aBMD: areal bone mineral density. Numbers in bold indicate statistical significance, concrete numbers are provided

Laboratory tests were performed at baseline (t0; at the time of the first dose or up to 3 months prior to the first Romosozumab administration), at 6 months (t1) after treatment initiation, and at 12 months (t2) (Fig. 1a). Serum and urine samples were analyzed to determine calcium homeostasis and bone turnover in our local laboratory as part of our clinical routine. Serum calcium, phosphate, alkaline phosphatase (ALP), glomerular filtration rate (GFR), bone-specific alkaline phosphatase (b-ALP), osteocalcin, 25-hydroxyvitamin D (25(OH)D), Procollagen type 1 N-terminal peptide (P1NP), C-terminal telopeptide of type I collagen (CTX), parathyroid hormone (PTH), and urinary deoxypyridinoline per creatinine in urine (DPD/Crea) were assessed.

### Dual-energy X-ray absorptiometry (DXA)

BMD parameters at baseline were collected using DXA scans performed within one year prior to the first administration of Romosozumab (Fig. 1a). For follow-up measurements, DXA scans were performed after the last administration of Romosozumab (t2—after 12 months). DXA (Lunar iDXA, GE Healthcare, Madison, WI, USA) scans were performed at the lumbar spine (L1–4) and proximal femora (femoral neck and total hip). Subsequently, the lowest T-score and the corresponding areal bone mineral density (aBMD) and Z-score were determined. Vertebrae affected by fractures or structural deformities were excluded from the analysis.

### High resolution peripheral quantitative computed tomography (HR-pQCT)

Bone microstructure parameters were assessed using HR-pQCT at t0, corresponding to the start of Romosozumab therapy, and at t2, after the last administration of Romosozumab (after 12 months) (Fig. 1a). Patients received either first- (XCT1) or second-generation (XCT2) HR-pQCT (XtremeCT and XtremeCT II, Scanco Medical AG, Brüttisellen, Switzerland) scans of the non-dominant distal radius and the opposite distal tibia, using the manufacturer’s standard *in-vivo* scan protocol for the respective HR-pQCT (XCT1: 59.4 kVp, 900 μA, 100 ms integration time, 82.0 μm isometric voxel size; XCT2: 68.0 kVp, 1,470 μA, 43 ms integration time, 60.7 μm isometric voxel size) [18]. The scan area begins at a fixed distance from the insertion point of the end plate of the distal radius or tibial plateau and extends proximally from there. For XCT1, the fixed distance is 9.5 mm at the radius and 22.5 mm at the tibia, whereas for XCT2 it is 9.0 mm and 22.0 mm, respectively. The scanning area covers 110 slices for XCT1 and 168 slices for XCT2, which corresponds to a total length of the scanning area of 9.0 mm and 10.2 mm, respectively. All scans were visually evaluated for motion artifacts using a standardized motion grading approach, and scans with excessive motion were excluded from further analysis, as recommended in the literature [19, 20].

Volumetric bone mineral density (vBMD) was quantified as total BMD (Tt.BMD, mg HA/cm3), cortical BMD (Ct.BMD, mg HA/cm3), and trabecular BMD (Tb.BMD, mg HA/cm3). Microarchitecture parameters included bone volume to total volume ratio (BV/TV), trabecular number (Tb. N, mm-1), trabecular thickness (Tb.Th, mm), trabecular separation (Tb.Sp, mm), and cortical thickness (Ct.Th, mm). To enable comparisons across devices, sex and age groups, HR-pQCT parameters were normalized, and the percentage median (% median) was calculated based on device-, age-, and sex-specific reference values for subsequent analyses [21, 22], thereby improving the comparability of results independent of scanner type, age, or sex. 

### Statistical analysis

For the statistical analysis, JASP 0.19.1 (University of Amsterdam, Netherlands) and GraphPad Prism 10.4.1 (GraphPad Software, San Diego, CA, USA) were used. Results were expressed as mean ± standard deviation (SD). The Shapiro–Wilk test was used to assess the normal distribution of the data. Group comparisons were performed using an unpaired two-tailed t-test for parametric data and the Mann–Whitney U test for nonparametric data. Repeated-measures ANOVA was applied where appropriate, with Holm’s post hoc test used for pairwise comparisons. For non-normally distributed repeated-measures data, Kendall’s W test was performed, followed by Conover’s post hoc test. Distribution differences in subgroups were tested using the chi-square test. Cohen’s d was calculated to determine the effect sizes (> 0.2 ≙ small, > 0.5 ≙ medium, > 0.8 ≙ large). P-values < 0.05 were considered to indicate a statistically significant difference between groups.

## Results

### Characterization of the study cohort

The baseline characteristics of the study cohort are shown in Table 1. The mean age was 53.6 ± 8.9 years for OI patients and 57.2 ± 5.8 years for OPO patients, with no significant difference between both groups (*p* = 0.444). Comorbidity burden assessed by the mean Charlson Comorbidity Index was comparable between both groups with 1.6 ± 0.9 in the OI group and 1.1 ± 0.6 in the OPO group, respectively (*p* = 0.347). Regarding mobility, all patients were able to mobilize without walking aids. Body weight and BMI were similar, however, OI patients had significantly lower body height compared to OPO patients (1.48 ± 0.07 m vs. 1.68 ± 0.07 m, *p* = 0.006). Two patients in both groups had received prior antiresorptive therapy (OI: 2/5; OPO: 2/10), with no significant difference between groups (*p* = 0.560). Spinal BMD parameters, including T-score, Z-score, and aBMD, did not differ significantly between the two groups. In contrast, femoral bone density differed significantly in both aBMD and Z-score with lower values in the OPO group (*p* = 0.014 and *p* = 0.007, respectively).

**Table 1:** Overview of the study cohort

Parameter	OI (*n* = 5)				OPO (*n* = 10)				
	Mean	SD	Min	Max	Mean	SD	Min	Max	p-value
Demographics									
Age (years)	53.6	8.9	42.0	66.0	57.2	5.8	47.0	66.0	0.444
Weight (kg)	53.8	10.2	43.0	67.0	65.6	16.5	38.0	94.0	0.132
Height (m)	1.48	0.07	1.38	1.55	1.68	0.07	1.60	1.77	**0.006**
BMI (kg/m^2^)	24.5	3.6	20.8	29.0	23.4	6.4	14.5	34.9	0.694
DXA									
Femoral BMD	0.763	0.037	0.734	0.805	0.659	0.078	0.532	0.776	**0.014**
Femoral T-score	−2.2	0.8	−3.3	−1.6	−2.6	0.6	−3.5	−1.7	0.365
Femoral Z-score	−0.8	0.1	−0.9	−0.9	−1.6	0.3	−1.9	−1.2	**0.007**
Spinal BMD	0.745	0.067	0.685	0.812	0.729	0.158	0.599	1.100	0.374
Spinal T-score	−3.6	0.5	−4.2	−3.1	−3.7	1.2	−5.0	−0.9	0.859
Spinal Z-score	−2.8	0.5	−3.2	−2.1	−2.6	0.8	−3.3	−0.7	0.810
Fragility fractures									
Vertebral fractures	8	7	1	17	3	3	1	9	0.172
Extravertebral fractures	13	4	7	18	2	2	0	7	**0.003**
Total fractures	19	3	16	23	5	4	1	12	** < 0.001**

Numbers in bold indicate statistical significance, concrete numbers are provided^1^

^1^*SD* standard deviation, *BMI* Body mass index, *DXA* dual-energy X-ray absorptiometry

The prevalence of extravertebral fractures was significantly higher in OI patients compared to OPO patients (13 ± 4 vs. 2 ± 2, *p* = 0.003), whereas the prevalence of vertebral fractures did not differ significantly (8 ± 7 vs. 3 ± 3, *p* = 0.172). Regarding adverse drug events, only mild injection side reactions were reported in both groups.

### Bone turnover parameters at baseline, 6 months, and 12 months of Romosozumab treatment

Biochemical laboratory analysis of the OI cohort revealed an increase in bone formation parameters at time points t1, and t2 (Table 2). Osteocalcin levels increased significantly between t0 and t1 (t0 vs t1: *p* = 0.005) and remained elevated at t2 (t0 vs t2: *p* = 0.008). P1NP also tended to rise during therapy, reaching significance in the overall repeated-measures ANOVA model (*p* = 0.047), although post hoc comparisons did not show significant differences. Serum b-ALP levels showed a similar upward trend from t0 to t2 without achieving statistical significance. No significant change was observed in the bone resorption markers CTX and DPD/Crea.

**Table 2:** Bone turnover markers in postmenopausal OI patients with Romosozumab treatment

		OI t0		OI t1		OI t2		p-value		
Laboratory parameters	Reference ranges	Mean	SD	Mean	SD	Mean	SD	t0 vs t1	t0 vs t2	t1 vs t2
Calcium (mmol/l)	2.18—2.60	2.45	0.12	2.42	0.10	2.48	0.12	0.886	0.886	0.886
Phosphate (mmol/l)	0.77—1.65	0.99	0.07	0.90	0.12	0.99	0.13	0.408	0.963	0.408
GFR (ml/min)	> 60	94.0	13.0	100.8	7.5	99.2	11.1	0.721	> 0.999	> 0.999
ALP (U/l)	46—116	97.8	13.6	106.8	12.7	104.0	9.8	> 0.999	> 0.999	> 0.999
Osteocalcin (µg/l)	5.4—59.1	18.7	2.9	23.4	2.9	22.9	3.4	**0.005**	**0.008**	0.580
25(OH)D (µg/l)	> 30	47.0	0.5	38.0	11.6	36.8	8.2	0.555	0.342	0.685
b-ALP (µg/l)	5.2—24.4	16.2	6.2	22.7	7.6	21.4	8.8	0.275	0.448	0.677
P1NP (µg/l)	24.0—134.0	28.9	9.2	46.8	19.0	44.1	18.4	0.137	0.246	0.591
CTX (µg/l)	0.183—1.060	0.175	0.087	0.222	0.108	0.178	0.100	0.128	0.940	0.359
PTH (ng/l)	17.4—80.1	40.9	17.5	45.8	22.5	36.2	9.1	> 0.999	> 0.999	> 0.999
DPD/Crea (nmol/mmol)	3—7	7	3	9	2	7	1	> 0.999	> 0.999	> 0.999

Laboratory biochemical analyses were performed at the baseline (t0), 6 months after the first dose (t1), and 12 months after Romosozumab treatment (t2) Reference ranges from the local laboratory are provided. Numbers in bold indicate statistical significance, concrete numbers are provided ^2^

^2 ^*SD *standard deviation, *ALP* alkaline phosphatase, *GFR *glomerular filtration rate, *25(OH)D* 25-hydroxyvitamin D (25(OH)D),* b-ALP* bone-specific alkaline phosphatase, *P1NP* procollagen type 1 N-terminal peptide, *CTX* C-terminal telopeptide of type I collagen, *DPD/Crea* deoxypyridinoline per creatinine in urine

The changes in laboratory parameters over a period of 12 months during Romosozumab treatment in the OPO patients are summarized in Table 3. A significant reduction in ALP was observed between t1 and t2 (*p* = 0.015), while osteocalcin levels showed no significant change. Serum concentrations of 25(OH)D increased significantly from baseline to month 6 (*p* < 0.001) under supplementation and remained within the reference range thereafter. b-ALP decreased significantly between 6 and 12 months (*p* = 0.002). P1NP showed a numerical increase after 6 months, followed by a decrease after 12 months, with a significant difference between 6 and 12 months (*p* = 0.023). CTX tended to decrease over time, although the changes did not reach statistical significance.

**Table 3:** Bone turnover markers in patients with postmenopausal osteoporosis (OPO) with Romosozumab treatment

		OPO t0		OPO t1		OPO t2		p-value		
Laboratory parameters	Reference ranges	Mean	SD	Mean	SD	Mean	SD	t0 vs t1	t0 vs t2	t1 vs t2
Calcium (mmol/l)	2.18—2.60	2.46	0.10	2.43	0.11	2.42	0.12	0.905	0.865	0.905
Phosphate (mmol/l)	0.77—1.65	1.06	0.13	0.99	0.10	1.00	0.21	0.818	> 0.999	> 0.999
GFR (ml/min)	> 60	93.4	10.5	94.2	6.8	92.6	9.7	> 0.999	> 0.999	> 0.999
ALP (U/l)	46—116	87.3	22.2	88.4	21.3	73.1	13.0	0.865	0.053	**0.015**
Osteocalcin (µg/l)	5.4—59.1	21.3	7.0	23.0	7.6	18.9	5.1	0.306	0.286	0.116
25(OH)D (µg/l)	> 30	33.4	6.3	47.1	11.6	43.9	10.8	** < 0.001**	** < 0.001**	0.284
b-ALP (µg/l)	5.2—24.4	14.1	4.4	15.3	3.6	12.9	3.6	0.576	0.576	**0.002**
P1NP (µg/l)	24.0—134.0	53.5	23.5	70.2	34.4	46.9	15.7	0.052	0.543	**0.023**
CTX (µg/l)	0.183—1.060	0.434	0.183	0.345	0.169	0.274	0.149	0.364	0.320	0.364
PTH (ng/l)	17.4—80.1	52.3	21.4	50.7	18.3	41.6	11.1	0.862	0.477	0.477
DPD/Crea (nmol/mmol)	3—7	7	4	8	3	7	2	> 0.999	> 0.999	> 0.999

Laboratory biochemical analyses were performed at the baseline (t0), 6 months after the first dose (t1), and 12 months after Romosozumab treatment (t2). Reference ranges from the local laboratory are provided. Numbers in bold indicate statistical significance, concrete numbers are provided ^3^

^3 ^*SD* standard deviation, *ALP* alkaline phosphatase, *GFR* glomerular filtration rate, *25(OH)D* 25-hydroxyvitamin D (25(OH)D), *b-ALP* bone-specific alkaline phosphatase, *P1NP *procollagen type 1 N-terminal peptide, *CTX* C-terminal telopeptide of type I collagen, *DPD/Crea* deoxypyridinoline per creatinine in urine

### Changes in areal bone mineral density (aBMD) after Romosozumab treatment

Following 12 months of treatment with Romosozumab, a significant increase in spinal aBMD parameters was observed in the OI group. The mean spinal T-score increased from −3.6 ± 0.5 to −2.9 ± 0.7 (*p* = 0.007), mean spinal Z-score from −2.8 ± 0.5 to −2.0 ± 0.7 (*p* = 0.019), and mean spinal aBMD from 0.739 ± 0.075 to 0.830 ± 0.099 g/cm^2^ (*p* = 0.007) (Fig. 1b). But no significant differences were observed in the femoral aBMD parameters in the OI group. In contrast, OPO patients showed significant improvements in both the spine and femoral aBMD parameters, with improvements in the mean values of spinal and femoral aBMD, T-score, as well as Z-score (Fig. 1c).

**Fig. 2:**
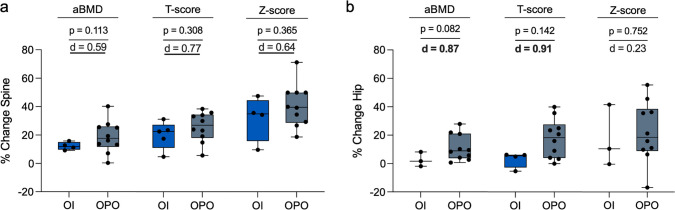
Percentage changes in bone mineral density in postmenopausal OI and postmenopausal osteoporosis patients treated with Romosozumab. Bone mineral density measurements of both spine (**a**) and hip (**b**) were performed within the first year before Romosozumab treatment and 12 months after Romosozumab treatment. The OI cohort is shown in blue and the OPO cohort in gray. aBMD: areal bone mineral density. Significant differences in the group comparisons are indicated by exact p-values with corresponding effect size Cohen’s d. Medium effect sizes (Cohen's d > 0.5) are marked by an underscore, and strong effect sizes (Cohen's d > 0.8) are marked in bold

When comparing the percentage change in aBMD parameters between OI patients and the OPO cohort after 12 months of Romosozumab treatment, no significant differences were found in either the spine or the hip between both groups (p > 0.05). However, a trend toward a greater increase in aBMD parameters was noted in the OPO group for both skeletal sites. At the spine, aBMD increased significantly in both groups, with a mean change of 12.2 ± 2.8% in the OI group and 18.8 ± 11.4% in the OPO group (*p* = 0.113). The increase in aBMD was less pronounced at the femur site, amounting to 2.7 ± 5.1% in the OI group and 11.2 ± 9.2% in the OPO group (*p* = 0.082). The effect size showed a medium effect for the percentage change at the spine (aBMD: d = 0.59, T-score: d = 0.77, Z-score: d = 0.64) (Fig. 2a), whereas it showed a predominantly large effect size for the aBMD parameters at the hip (aBMD: d = 0.87, T-score: d = 0.91, Z-score: d = 0.23) (Fig. 2b).

In a descriptive subgroup analysis of the OI cohort comparing treatment-naïve and previously treated patients, treatment-naïve patients had a greater increase in lumbar spine and hip aBMD. These descriptive findings should be interpreted with caution due to the small sample size. However, the trend suggests a potentially stronger aBMD response in treatment-naïve patients, which needs to be confirmed in larger studies.

### Assessment of peripheral bone microstructure using HR-pQCT

HR-pQCT analysis of both the radius and tibia showed no significant changes in the measured parameters between t0 and t2 for the OI group (Table 4). However, there were trends towards improvements in bone microstructure with moderate to high effect sizes in both trabecular and cortical microstructure at the distal radius and tibia (p > 0.05, effect sizes between 0.26 and 1.07).

**Table 4:** Longitudinal changes in bone microstructure measured by HR-pQCT in postmenopausal classical OI patients and in patients with postmenopausal osteoporosis (OPO) receiving Romosozumab treatment

	OI t0		OI t2				OPO t0		OPO t2			
Parameter	Mean	SD	Mean	SD	p value	Cohen’s d	Mean	SD	Mean	SD	p-value	Cohen’s d
HR-pQCT Radius												
Tt.BMD (%median)	86.5	8.2	84.8	10.3	0.578	0.31	69.4	12.5	74.0	12.6	0.080	0.67
Tb.BMD (%median)	55.5	9.1	67.4	20.6	0.379	0.52	62.0	18.5	67.6	18.4	0.250	0.57
BV/TV (%median)	69.8	8.3	82.0	21.6	0.625	0.53	69.0	15.7	75.8	13.6	0.110	**0.85**
Tb.N (%median)	52.7	13.6	51.5	13.8	0.598	0.29	70.2	20.9	71.5	27.0	0.426	0.42
Tb.Th (%median)	109.6	7.8	125.9	34.6	0.875	0.26	98.3	9.3	100.7	10.4	0.359	0.47
Tb.Sp (%median)	217.6	77.7	236.5	108.7	0.608	0.29	164.4	62.6	175.9	95.1	0.652	0.25
Ct.BMD (%median)	93.8	3.9	90.3	9.6	0.608	0.29	88.0	5.8	89.7	4.2	0.491	0.02
Ct.Th (%median)	95.0	10.6	97.7	23.2	0.796	0.26	78.9	15.4	80.4	15.8	0.947	0.24
HR-pQCT Tibia												
Tt.BMD (%median)	67.3	11.9	68.8	12.1	0.074	**1.07**	70.3	14.7	75.1	16.6	**0.008**	**1.44**
Tb.BMD (%median)	46.1	12.7	47.1	12.9	0.188	**1.08**	69.0	18.2	72.4	20.4	0.133	0.56
BV/TV (%median)	62.2	10.0	63.9	9.5	0.081	**1.04**	75.1	18.0	78.7	19.2	0.077	0.68
Tb.N (%median)	32.4	21.8	32.3	20.9	0.830	0.10	76.1	17.5	78.8	16.9	0.571	0.20
Tb.Th (%median)	121.6	12.0	119.9	7.0	0.552	0.29	99.1	16.9	100.7	17.9	0.779	0.16
Tb.Sp (%median)	436.7	189.7	432.2	183.1	0.504	0.33	144.4	42.0	136.5	35.5	0.366	0.32
Ct.BMD (%median)	87.4	6.5	88.3	8.1	0.692	0.20	88.3	4.3	90.2	4.6	0.345	0.33
Ct.Th (%median)	85.4	11.2	87.3	12.4	0.282	0.56	80.0	12.3	83.7	10.4	0.132	0.56

HR-pQCT measurements were performed for the baseline (t0) and after 12 months undergoing Romosozumab treatment (t2). HR-pQCT results for the distal radius and tibia were compared with the median device-, age-, and gender-specific reference values to ensure better comparability. Significant differences in the group comparisons are indicated by exact p-values with corresponding effect size Cohen’s d. Medium effect sizes (Cohen's d > 0.5) are marked by an underscore, and strong effect sizes (Cohen's d > 0.8) are marked in bold ^4^

^4^*SD* standard deviation, *HR-pQCT* high-resolution peripheral quantitative computed tomography, *Tt.BMD* total BMD, *HA* hydroxyapatite, *Tb.BMD* trabecular BMD, *BV/TV* bone volume to tissue volume, *Tb.N* trabecular number, *Tb.Th* trabecular thickness, *Tb.Sp *trabecular separation, *Ct.BMD* cortical BMD, *Ct.Th* cortical thickness

In the OPO group, HR-pQCT analysis revealed also trends toward improved trabecular and cortical parameters (Table 4). At the radius, Tt.BMD showed an increase from 69.4 ± 12.5% to 74.0 ± 12.6% (*p* = 0.080, d = 0.67), and BV/TV exhibited a similar rise from 69.0 ± 15.7% to 75.8 ± 13.6% (*p* = 0.110, d = 0.85). At the tibia, Tt.BMD increased significantly (70.3 ± 14.7% to 75.1 ± 16.6%, *p* = 0.008, d = 1.44), with moderate but non-significant improvements in the remaining bone microstructure parameters.

## Discussion

Romosozumab is a humanized monoclonal anti-sclerostin antibody that had been established as a highly effective osteoanabolic treatment for postmenopausal women with severe osteoporosis. The dual effect, stimulating bone formation while inhibiting bone resorption, has been associated with substantial gains in aBMD of up to 16.9% at the lumbar spine [23] as well as 6.9% at the total hip [13], which was associated with a reduced risk of fractures [14, 24–26]. Whereas treatment options increase for postmenopausal osteoporosis patients over the last decades, pharmacological treatment of adult patients with OI remains a challenge. However, recently Setrusumab, another monoclonal anti-sclerostin antibody, has been developed and is currently under clinical investigation to specifically improve treatment for OI patients. [15, 16].

Here, we investigated the effect of 12 months Romosozumab treatment in postmenopausal women with OI type I and IV with regard to bone mass and structure as well as bone turnover parameters in comparison to postmenopausal osteoporosis patients treated with Romosozumab. This is of high interest also since only a limited number of studies have investigated adults with OI using HR-pQCT [27–29], and evidence regarding Romosozumab treatment in OI today remains confined to very few case reports [30–32].

Using Romosozumab, in our OI cohort we observed a significant increase in the osteoblast marker osteocalcin, along with a non-significant increase in bone formation marker P1NP and b-ALP, in line with literature [16]. While the dual mechanism of anti-sclerostin antibodies in bone turnover parameters has been reported in patients with osteogenesis imperfecta [15, 16], markers of bone resorption (CTX and DPD/Crea) showed little change during treatment in our cohort. A comparable pattern was reported in the Phase 2b trial of Setrusumab in patients with OI, where bone formation markers increased markedly during the first months of treatment and gradually returned toward baseline after 12 months [15]. These findings suggest that anti-sclerostin treatment in OI patients induces an early anabolic response, primarily through increased bone formation without a sustained suppression of bone resorption. In the OPO cohort we observed a laboratory pattern in line with Cosman et al. [13], although the changes did not reach statistical significance given the small group size. Thus, the bone dual action anabolic and antiresorptive turnover profile is more prominent in postmenopausal osteoporosis patients than in OI patients.

First studies have shown a significant increase in aBMD using anti-sclerostin-antibodies in OI. This raised the question of the extent of an aBMD increase in postmenopausal patients with OI treated with Romosozumab. In our OI cohort, a significant increase in spinal aBMD was observed for both, OI and OPO patients following 12 months of Romosozumab treatment. At the hip, the anabolic effect was less pronounced especially in the OI group, consistent with site-specific treatment responses favoring the spine [33]. Although not reaching significance, medium to large effect sizes were observed for changes in aBMD comparing OI to OPO patients, suggesting that OPO patients experience a greater increases in aBMD. In this context, OPO patients had significantly lower femoral aBMD at baseline, which may partly explain the greater increase in hip aBMD. Consistent with Gielen et al., who demonstrated that lower baseline aBMD in postmenopausal women receiving romosozumab was independently associated with a greater aBMD response in the total hip [33]. Furthermore, differences in the underlying pathophysiology between the two groups may also contribute to differences in response to sclerostin inhibition. The relatively small sample size of the OI cohort may have limited the statistical power to detect changes in aBMD. Nonetheless, the increase in spinal aBMD observed in our OI cohort was greater compared to other osteoanabolic agents used in patients with OI [5, 7]. For instance, 18 months with teriparatide treatment increased spinal aBMD by 7.6% in OI type I patients [5] compared to 12.2% in our OI cohort. The significant increase in aBMD at the spine in postmenopausal OI patients observed in this study is encouraging. However, the observed increase in lumbar spine aBMD should be interpreted with caution as improvements in aBMD may not necessarily translate into fracture risk reduction in OI patients. In this context, the very recently published TOPAZ study demonstrated a significant increase in lumbar spine aBMD following treatment with teriparatide and zoledronic acid in adults with OI, whereas no significant decrease in fracture incidence was observed [34]. Furthermore, neither female sex nor age above 50 were associated with a reduction in fracture rate despite spine BMD increase. Thus, it remains unclear at this point whether increases in aBMD with anti-sclerostin therapy translates into clinically significant fracture risk reduction, and further prospective long-term studies are urgently needed.

Beyond the assessment of bone mineral density, the qualitative evaluation of bone microstructure has gained increasing importance in understanding bone strength and fracture risk [35, 36]. In OI patients, previous studies have demonstrated marked deficits of the bone structure in both, the trabecular and cortical compartment [27–29, 37]. After 12 months of Romosozumab treatment, no statistically significant changes in microstructural parameters at both radius and tibia were observed in the OI group. However, several parameters showed trends toward improvement, with moderate to high effect sizes indicating potential benefits, especially for the trabecular bone. Similar findings were observed in the Setrusumab phase 2b trial, but with significant improvements in estimated bone strength and microstructural parameters [15]. The absence of statistical significance in our study may be attributed to the small sample size (n = 5), which limits the statistical power to detect microstructural treatment effects. Overall, an anabolic effect of Romosozumab on spinal aBMD in OI patients as well as improvements of trabecular bone microstructure is emphasized by our results.

This study has limitations that should be acknowledged. The retrospective design inherently limits the ability to control for confounding variables and to establish causal relationships. The ambulation status was not systematically assessed due to the retrospective study design and should be included in future prospective studies. The small sample size reduces the statistical power to detect significant effects of Romosozumab on bone microarchitecture and turnover markers. Furthermore, sample size and observation period were not sufficient to assess fractures or fracture risk. No patients with OI type III were included in the study and although only patients with OI type I and IV were included, no differentiation for genetic variants (*COL1A1/A2* or quantitative/qualitative) was performed, which may have masked potential differences in collagen-related bone phenotype and treatment response. Previous studies have reported differences in bone microstructure after genetic stratification [28, 29], which further underscores the need for a systematic evaluation in larger prospective studies.

## Conclusion

The observed increase in spinal aBMD supports the potential of anti-sclerostin therapy as a treatment option for postmenopausal patients with OI. However, the clinical relevance of increased aBMD with regard to fracture risk reduction remains uncertain. No significant changes were observed in aBMD at the hip and bone microstructure after 12 months of Romosozumab treatment in our OI cohort, which might be due to the limited group size. These findings emphasize the need for larger studies in adults with OI in order to improve the understanding of anti-sclerostin treatment effects on bone mass, structure and quality and to optimize therapeutic strategies.

## Data Availability

The relevant data is presented within the manuscript. Further data can be requested from the corresponding author upon reasonable request although patient data may be restricted for privacy and data protection reasons.
